# Effects of Postprandial Blood Pressure on Gait Parameters in Older People

**DOI:** 10.3390/nu8040219

**Published:** 2016-04-13

**Authors:** Shailaja Nair, Renuka Visvanathan, Diana Piscitelli

**Affiliations:** 1 Aged and Extended Care Services, The Queen Elizabeth Hospital, Woodville, South Australia 5011, Australia; shailaja.nair@sa.gov.au (S.N.); renuka.visvanathan@adelaide.edu.au (R.V.); 2 Adelaide Geriatrics Training and Research with Aged Care (G-TRAC) Centre, School of Medicine, University of Adelaide, Adelaide, South Australia 5000, Australia; 3 School of Health Sciences, University of South Australia, Adelaide, South Australia 5000, Australia

**Keywords:** glucose, postprandial hypotension, older, elderly, aging, gait, blood pressure, walk

## Abstract

Postprandial hypotension (PPH), a fall in systolic blood pressure (SBP) within 2 h of a meal, may detrimentally affect gait parameters and increase the falls risk in older people. We aimed to determine the effects of postprandial SBP on heart rate (HR), gait speed, and stride length, double-support time and swing time variability in older subjects with and without PPH. Twenty-nine subjects were studied on three days: glucose (“G”), water and walk (“WW”), glucose and walk (“GW”). Subjects consumed a glucose drink on “G” and “GW” and water on “WW”. The “G” day determined which subjects had PPH. On “WW” and “GW” gait was analyzed. Sixteen subjects demonstrated PPH. In this group, there were significant changes in gait speed (*p* = 0.040) on “WW” and double-support time variability (*p* = 0.027) on “GW”. The area under the curve for the change in gait parameters from baseline was not significant on any study day. Among subjects without PPH, SBP increased on “WW” (*p* < 0.005) and all gait parameters remained unchanged on all study days. These findings suggest that by changing gait parameters, PPH may contribute to an increased falls risk in the older person with PPH.

## 1. Introduction

Falls are common in older people, with a prevalence of 30% and 50% among community dwelling individuals and older people living in residential care facilities, respectively [1,2,3]. A major consequence of falls is fractures, which pose a considerable public health issue due to the association with detrimental physical and psychological morbidity [4,5], as well as significant health care costs [6]. Falls risk factors are typically multifactorial [7] and include postprandial hypotension (PPH) and gait impairments [5,8].

PPH is a condition that is defined as a 20 mmHg reduction in systolic blood pressure (SBP) or a decline in SBP to less than 90 mmHg from a pre-ingestion SBP of greater than 100 mmHg, occurring within 2 h of meal commencement [9,10]. There is a strong association between PPH and falls in older people. PPH is present in 25% of older inpatients with a history of falls [11], 50% of older persons with unexplained falls occurring within 2 h of a meal [9] and in 58% of older patients seen in a falls outpatient clinic [12].

There is a strong association between gait impairment and falls, such that when the former is evident, the risk of falls is increased [5,7,13,14]. In a review of 12 studies evaluating falls risk factors among older people, gait impairment was identified as the second largest contributory cause for falls [5]. Moreover, a systematic review and meta-analysis of 74 studies investigating risk factors for falls among community-dwelling older people found that gait impairment ranked as the second strongest falls risk factor [14]. Specific gait parameters that have been robustly associated with falls are a decrease in gait speed and an increase in the variability of stride length, double-support time and swing time [15,16,17].

Previous studies have determined that a relationship exists between: (a) falls and PPH [9,11,12,18]; and (b) falls and gait impairment [5,7,13,14]. However, it is uncertain if the decline in SBP evident in people with PPH has an effect on gait parameters which may then predispose the older person to falls. Hence, the aim of this study was to determine if a postprandial decline in SBP affects gait speed, stride length variability, double-support time variability or swing time variability in individuals with and without PPH.

## 2. Experimental Section

### 2.1. Subjects

Twenty-nine older subjects (18 female (mean age 77.1 ± 5.4 years), 11 male (mean age 74.7 ± 3.9 years)) with and without PPH were recruited from geriatrician clinics at The Queen Elizabeth Hospital, from lists of research subjects that had previously participated in research studies or via community events related to the Healthy Ageing Program undertaken by the Adelaide Geriatrics Training and Research with Aged Care (G-TRAC) Centre. Recruitment occurred through a number of methods in order to minimize selection bias, and consistent criteria was used to recruit and enroll all subjects into the study. Subjects aged 65 years and above were included in the study with the exception of those who had a history of significant cardiovascular disease (myocardial infarction less than three months earlier, clinical coronary artery disease or symptomatic congestive heart failure), significant respiratory disease, renal impairment, epilepsy, dysphagia, chronic alcohol abuse or excessive cigarette smoking (more than 10 cigarettes a day). Subjects withheld usual medication on the morning of the study days.

This research study was approved by the Human Research Ethics Committee of The Queen Elizabeth Hospital/Lyell McEwin Hospital/Modbury Hospital, Adelaide, Australia (protocol number 2011011; 1 November 2011). Each subject provided written, informed consent before the commencement of the first study day. All experiments were carried out in accordance with the Declaration of Helsinki.

### 2.2. Experimental Design

Each subject was studied on three randomized study days, separated by a minimum of 72 h. To minimize bias, an online randomization program [19] was used. On each study day, subjects underwent an overnight fast (10 h for solids and 6 h for liquids) and in the preceding 24 h refrained from alcohol and caffeine containing beverages, and smoking for 12 h.

The subjects attended the Basil Hetzel Institute at The Queen Elizabeth Hospital at 0830 h on each day and upon arrival were seated comfortably in a chair. An automated blood pressure cuff was placed around the right arm and following a 10 min rest period, at *t* = 0 min, subjects ingested a study drink. On two of the study days, (glucose (“G”) and glucose and walk (“GW”)), the drink consisted of 50 g glucose (glucose monohydrate, Fluka Analytical, Sigma-Aldrich Pty Ltd., Castle Hill, NSW, Australia) dissolved in water (total volume of the drink 200 mL). On the other study day, the drink consisted of 200 mL of water alone (“WW”). The purpose of the “G” day was to establish which subjects had PPH so that the subjects could be grouped; it has been well established that ingestion of a glucose drink induces a rapid fall in SBP, the magnitude of which fulfills the definition of PPH [9], *i.e.*, a 20 mmHg fall in SBP after glucose ingestion [9]. On one day, cardiovascular autonomic nerve function and orthostatic blood pressure were evaluated before the commencement of the study [20].

### 2.3. Assessment of Subject Health Status

Before the commencement of the first study day, subjects were interviewed by a medical doctor using a standardized questionnaire consisting of questions relating to demographic variables, medical history, medication use, history of falls and fear of falls. This information was obtained via telephone, with the use of a questionnaire sent by mail and by directly interviewing the subject in person on the first study day. Global assessment of health status was assessed by calculating the Charlson Comorbidity Index (CCI), a measure of comorbidity which predicts the one-year mortality rate for a patient by taking into account both the number and severity of 19 pre-defined comorbid conditions [21]. Each condition is assigned a score of 1, 2, 3 or 6, depending on the mortality risk associated with the condition. The scores are summed and a total score is derived. The one-year mortality rates for the different scores are: “0”: 12%; “1–2”: 26%; “3–4”: 52%; and “greater than or equal to “5””: 85% [21].

Fear of falls was assessed using the Falls Efficacy Scale International, a widely accepted tool for assessing concern about falling [22], which has been validated in an Australian population [23]. It is a self-reporting questionnaire, providing information on the level of concern about falls for a range of 16 activities of daily living scored on a four-point scale anchored at one end by 1 = not at all concerned and the other end by 4 = very concerned. A score of 23 and above indicates a high concern about falling [23].

### 2.4. Blood Pressure and Heart Rate Measurements

SBP and heart rate (HR) were measured using an automated oscillometric blood pressure monitor (Spacelabs Ultralite 24 hour ABP, JLM Accutek Health Care, Homebush, NSW, Australia). On all study days, “baseline” SBP and HR (*t* = 0 min) were calculated as the mean of measurements taken at *t* = −9, −6, and −3 min before ingestion of the drink. SBP and HR were measured every 6 min between *t* = 0–60 min and then every 15 min between *t* = 60–120 min.

### 2.5. Gait Parameters Acquisition and Analysis

Gait parameters were measured using the GAITRite^®^ walkway system (CIR Systems Inc., Franklin, NJ, USA). This system consisted of a 6 m portable walkway with a series of sensor pads activated by mechanical pressure [24]. Data from the sensors were collected by a series of on-board processors and transferred and stored on a computer. Previous studies have demonstrated that the GAITRite^®^ measures gait parameters with strong concurrent validity and test-retest reliability in older people [24,25,26,27]. Gait speed and variability in stride length, double-support time and swing time were recorded at each subject’s self-selected walking speed, *i.e.*, at a pace deemed comfortable by each individual subject. These parameters were selected as they have been identified as the most clinically relevant in terms of association with falls in older people [13,15,16,17]; a decline in gait speed [13,15,16,19] and increased variability in stride length, double-support time and swing time have been identified as risk factors for future falls [17]. In this study, gait speed was derived by dividing the distance walked by the ambulation time [28]. Stride length was determined by the anterior-posterior distance between the heels of two consecutive footprints of the same foot [29,30]. Double-support time was assessed as the time that both feet were on the ground simultaneously [29,30]. Swing time was measured as the time elapsed between the last contact of the current footfall to the initial contact of the next footfall of the same foot [29,30]. Variability, which refers to the intra-individual stride-to-stride fluctuations in gait characteristics [29], was calculated by dividing the standard deviation by the mean of the relevant gait parameter and multiplying this by 100 ((standard deviation/mean) × 100) [29].

Gait was analyzed on the “WW” and “GW” days only. At *t* = −20 min, subjects undertook two practice walks to familiarize themselves with the protocol and GAITRite^®^, followed by two test walks. Subsequent gait analysis every 30 min between *t* = 30–120 min involved two test walks only (*i.e.*, no practice walks). Gait measurements were calculated as the mean of the two walks [31]. To ensure a consistent walking pace, subjects commenced and completed walking approximately 2 m before and after the walkway [31,32], *i.e.*, total walking distance was 10 m.

### 2.6. Cardiovascular Autonomic Function

Autonomic nerve function was assessed using standardized cardiovascular reflex tests [33,34]. Parasympathetic function was determined by the variation (R-R interval) of HR during deep breathing and response to standing (30:15 ratio). Sympathetic function was assessed by the fall in SBP in response to standing. Each of the test results was scored according to age-adjusted predefined criteria as 0 = normal, 1 = borderline and 2 = abnormal for a total maximum score of 6. A score > 3 was considered to indicate autonomic dysfunction [33,34].

### 2.7. Orthostatic Blood Pressure

Orthostatic hypotension was determined by measuring SBP in the supine position and then after standing for one and three min. Orthostatic hypotension was diagnosed as a decline in SBP of 20 mmHg or more and/or a decline in diastolic blood pressure of 10 mmHg or more between the supine and standing positions, evident on at least one of the time points [35].

### 2.8. Statistical Analysis

Power calculations from preliminary data from the first five subjects with PPH enrolled in this study determined that to detect a difference of 10 cm/s in gait speed, 16 subjects with PPH were required to provide an 80% power, at the 0.05 significance level. A value of 10 cm/s was chosen as this has been demonstrated to be clinically relevant [36]. SBP, HR, gait speed, stride length variability, double-support time variability and swing time variability were analyzed as changes from baseline. One-way analysis of variance was used to analyze the effects of time on these variables.

The maximum changes in SBP, HR, gait speed, stride length variability, double-support time variability and swing time variability were defined as the greatest mean changes from baseline in each subject at any given time point for each study day, and is reported where a significant change from baseline is demonstrated. The area under the curve (AUC) between *t* = 0–120 min was calculated using the trapezoidal rule and analyzed by paired *t*-test to evaluate a “treatment” effect for SBP, HR, gait speed, stride length variability, double-support time variability and swing time variability.

Subjects’ characteristics were summarized using means and standard deviations, or frequencies and percentages, as appropriate. Comparisons between the characteristics of subject groups were performed using the chi-squared test and independent-samples *t*-test. All analyses were performed using SPSS Version 20 (SPSS Inc., Chicago, IL, USA). Data are presented as means ± standard deviation (SD). A *p* value < 0.05 was considered statistically significant in all analyses. Analyses were performed with the assistance of a professional biostatistician.

## 3. Results

### 3.1. Subject Characteristics

All recruited subjects completed the research study, which was well tolerated with no adverse effects reported. Sixteen of the 29 subjects demonstrated PPH, defined as a decline of 20 mmHg in SBP occurring within 2 h of consuming the study drink [9,10] on the “G” day (Table 1).

Subjects’ characteristics are summarized in Table 2. When compared with subjects without PPH, those subjects with PPH had a significantly higher (*p* = 0.006) CCI (*i.e.*, indicating that this group had more co-morbidities), and significantly more subjects with hypertension (*p* = 0.034) and increased antihypertensive use (*p* = 0.017). Furthermore, amongst this group, four had orthostatic hypotension and of these, two also had definite autonomic dysfunction. In contrast, among subjects without PPH, only two had orthostatic hypotension and none had autonomic dysfunction. There were no significant differences between the two groups of subjects for all other variables in the assessment of health status.

### 3.2. Baseline Gait Parameters

Table 3 summarizes baseline gait speed, and variability in stride length, double-support time and swing time. There were no significant differences between the two groups of subjects for these baseline parameters.

### 3.3. Systolic Blood Pressure

#### 3.3.1. Subjects with PPH

**Figure 1:**
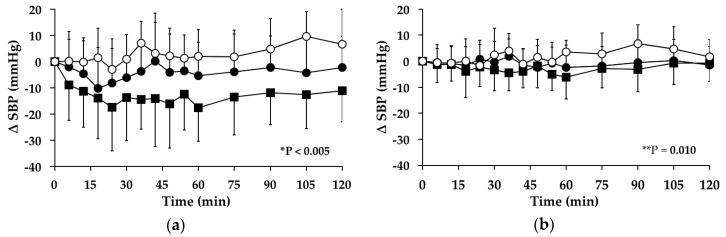
Mean change (△) in systolic blood pressure (SBP) on “G” (■), “WW” (○) and “GW” (●) days in subjects: (**a**) with postprandial hypotension (PPH); and (**b**) without PPH. Data are mean ± standard deviation (SD) represented by vertical bars. * *p* < 0.005 and ** *p* = 0.010 for the area under the curve (AUC) for the change in SBP from baseline between the study days.

There was a significant fall in SBP over time between *t* = 0–120 min on the “G” day (*p* < 0.005) and no signficant change on the “GW” (*p* = 0.397) or “WW” (*p* = 0.067) days. The mean maximum fall, and time of fall, in SBP on the “G” day were 26.69 ± 8.43 mm Hg at 54.0 ± 36.0 min (Table 1). Between *t* = 0–120 min there was a significant treatment effect for the AUC for the change in SBP from baseline between the study days (*p* < 0.005). During this time, compared to the “G” day, SBP was significantly greater during the “GW” day (95% CI = −1741–−271, *p* = 0.007) and “WW” day (95% CI = −2469–−1259, *p* < 0.005) (Figure 1a). At *t* = 120 min, SBP was significantly less than baseline on the “G” day (*p* = 0.001) and there was no difference on the “GW” (*p* = 0.446) or the “WW” (*p* = 0.080) days.

#### 3.3.2. Subjects without PPH

There were no significant changes in SBP over time between *t* = 0–120 min on the “G” (*p* = 0.108) or “GW” (*p* = 0.683) days. There was a significant increase in SBP over time between *t* = 0–120 min on the “WW” day (*p* < 0.005). The mean maximum increase, and time of increase, in SBP on the “WW” day were 6.62 ± 13.51 mmHg at 42 ± 29 min. Between *t* = 0–120 min there was a significant treatment effect for the AUC for the change in SBP from baseline between the study days (*p* = 0.010). During this time, SBP was significantly greater during the “WW” day (95% CI = −951–−157, *p* = 0.007) than the “G” day but not during the “GW” day (95% CI = −562–−951, *p* = 1.000) (Figure 1b). At *t* = 120 min, SBP was not different from baseline on the “G” (*p* = 0.679), “WW” (*p* = 0.410) or “GW” (*p* = 0.510) days.

### 3.4. Heart Rate

#### 3.4.1. Subjects with PPH

**Figure 2:**
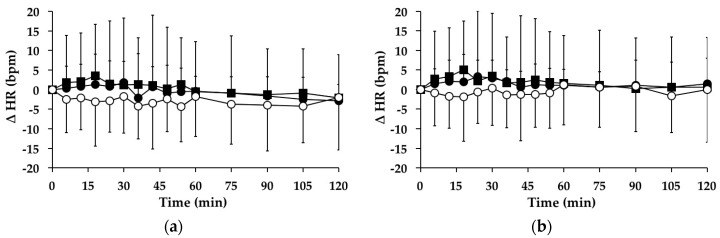
Mean change (△) in heart rate (HR) on “G” (■), “WW” (○) and “GW” (●) days in subjects: (**a**) with postprandial hypotension (PPH); and (**b**) without PPH. Data are mean ± standard deviation (SD) represented by vertical bars.

There were no significant changes in HR over time between *t* = 0–120 min on the “G” (*p* = 0.667), “GW” (*p* = 0.271) or “WW” (*p* = 0.465) days. Between *t* = 0–120 min, there was no significant treatment effect for the AUC for the change in HR from baseline between the study days (95% CI = −218–419, *p* = 0.220) (Figure 2a). At *t* = 120 min, HR was not significantly different than baseline on the “G” (*p* = 0.117), “GW” (*p* = 0.307) or “WW” (*p* = 0.197) days.

#### 3.4.2. Subjects without PPH

There were no significant changes in HR over time between *t* = 0–120 min on the “G” (*p* = 0.567), “GW” (*p* = 0.905) or “WW” (*p* = 0.755) days. Between *t* = 0–120 min, there was no significant treatment effect for the AUC for the change in HR from baseline between the study days (95% CI = 258–332, *p* = 0.116) (Figure 2b). At *t* = 120 min, HR was not significantly different than baseline on the “G” (*p* = 0.646), “GW” (*p* = 0.527) or “WW” (*p* = 1.000) days.

### 3.5. Gait Speed

#### 3.5.1. Subjects with PPH

**Figure 3:**
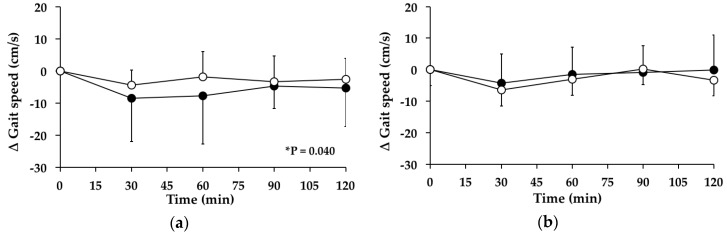
Mean change (△) in gait speed on “WW” (○) and “GW” (●) days in subjects: (**a**) with postprandial hypotension (PPH); and (**b**) without postprandial PPH. Data are mean ± standard deviation (SD) represented by vertical bars. * *p* = 0.040 for the change in gait speed over time on the “WW” day.

There was no signficant change in gait speed on the “GW” day (*p* = 0.071). However, there was a significant change in gait speed over time between *t* = 0–120 min on the “WW” day (*p* = 0.040). The mean maximum change, and time of change, in gait speed on the “WW” day were −4.34 ± 10.01 cm/s at 60.0 ± 33.0 min. Between *t* = 0–120 min, there was no significant treatment effect for the AUC for the change in gait speed from baseline between the study days (95% CI = −289–930, *p* = 0.280) (Figure 3a). At *t* = 120 min, gait speed was not different from baseline on the “WW” day (*p* = 0.126).

#### 3.5.2. Subjects without PPH

There were no significant changes in gait speed over time between *t* = 0–120 min on the “WW” (*p* = 0.225) or “GW” (*p* = 0.308) days. Between *t* = 0–120 min, there was no significant treatment effect for the AUC for the change in gait speed from baseline between the study days (95% CI = −289–930, *p* = 0.280) (Figure 3b). At *t* = 120 min, gait speed was not different from baseline on the “WW” (*p* = 0.169) or “GW” days (*p* = 0.973).

### 3.6. Stride Length Variability

#### 3.6.1. Subjects with PPH

**Figure 4:**
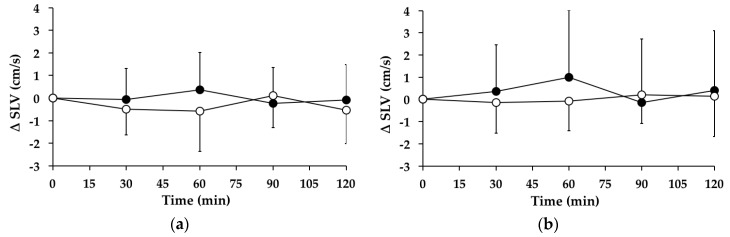
Mean change (△) in stride length variability (SLV) on “WW” (○) and “GW” (●) days in subjects: (**a**) with postprandial hypotension (PPH); and (**b**) without PPH. Data are mean ± standard deviation (SD) represented by vertical bars.

There were no significant changes in stride length variability over time between *t* = 0–120 min on the “GW” (*p* = 0.344) or “WW” (*p* = 0.402) days. Between *t* = 0–120 min, there was no significant treatment effect for the AUC for the change in stride length variability from baseline between the “WW” and “GW” days (95% CI = −132–69, *p* = 0.513) (Figure 4a). At *t* = 120 min, stride length variability was not different from baseline on the “WW” (*p* = 0.156) or “GW” (*p* = 0.825) days.

#### 3.6.2. Subjects without PPH

There were no significant changes in stride length variability over time between *t* = 0–120 min on the “WW” (*p* = 0.938) or “GW” (*p* = 0.733) days. Between *t* = 0–120 min, there was no significant treatment effect for the AUC for the change in stride length variability from baseline between the “WW” and “GW” days (95% CI = −132–69, *p* = 0.513) (Figure 4b). At *t* = 120 min, stride length variability was not different from baseline on the “WW” (*p* = 0.789) or “GW” (*p* = 0.599) days.

### 3.7. Double-Support Time Variability

#### 3.7.1. Subjects with PPH

**Figure 5:**
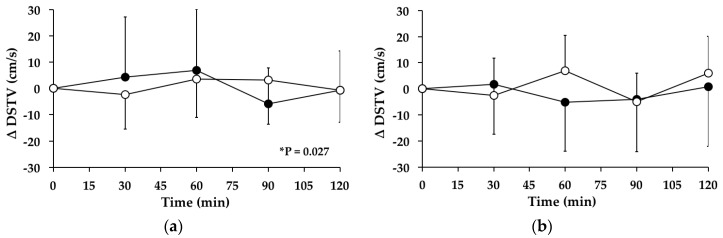
Mean change (△) in double-support time variability (DSTV) on “WW” (○) and “GW” (●) days in subjects: (**a**) with postprandial hypotension (PPH); and (**b**) without PPH. Data are mean ± standard deviation (SD) represented by vertical bars. * *p* = 0.027 for the change in DSTV over time on the “GW” day.

There was a significant change (*p* = 0.027) in double-support time variability over time between *t* = 0–120 min on the “GW” day, but not on the “WW” day (*p* = 0.339). The mean maximum change, and time of change, in double-support time variability on the “GW” day was 10.83 ± 25.03 cm/s at 60.0 ± 28.0 min. Between *t* = 0–120 min, there was no significant treatment effect for the AUC for the change in double-support time variability from baseline between the “WW” and “GW” days (95% CI = −666–813, *p* = 0.835) (Figure 5a). At *t* = 120 min, double-support time variability was not different from baseline on the “WW” (*p* = 0.830) or “GW” (*p* = 0.853) days.

#### 3.7.2. Subjects without PPH

There were no significant changes in double-support time variability over time between *t* = 0–120 min on the “WW” (*p* = 0.257) or “GW” (*p* = 0.222) days. Between *t* = 0–120 min, there was no significant treatment effect for the AUC for the change in double-support time variability from baseline between the “WW” and “GW” days (95% CI = −648–1337, *p* = 0.464) (Figure 5b). At *t* = 120 min, double-support time variability was not different from baseline on the “WW” (*p* = 0.154) or “GW” (*p* = 0.900) days.

### 3.8. Swing Time Variability

#### 3.8.1. Subjects with PPH

**Figure 6:**
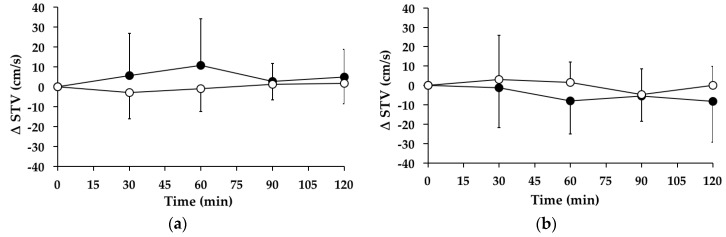
Mean change (△) in swing time variability (STV) on “WW” (○) and “GW” (●) days in subjects: (**a**) with postprandial hypotension (PPH); and (**b**) without PPH. Data are mean ± standard deviation (SD) represented by vertical bars.

There were no significant changes in swing time variability over time between *t* = 0–120 min on the “WW” (*p* = 0.798) or “GW” (*p* = 0.342) days. Between *t* = 0–120 min, there was no significant treatment effect for the AUC for the change in swing time variability from baseline between the “WW” and “GW” days (95% CI = −1331–193, *p* = 0.133) (Figure 6a). At *t* = 120 min, swing time variability was not different from baseline on the “WW” (*p* = 0.556) or “GW” (*p* = 0.186) days.

#### 3.8.2. Subjects without PPH

There were no significant changes in swing time variability between *t* = 0–120 min on the “WW” (*p* = 0.363) or “GW” (*p* = 0.594) days. Between *t* = 0–120 min, there was no significant treatment effect for the AUC for the change in swing time variability from baseline between the “WW” and “GW” days (95% CI = −318–1323, *p* = 0.207) (Figure 6b). At *t* = 120 min, swing time variability was not different from baseline on the “WW” (*p* = 0.995) or “GW” (*p* = 0.188) days.

## 4. Discussion

This is the first study to evaluate the effects of postprandial SBP on gait parameters known to be associated with increased risk of falls [15,16,17], in older individuals with and without PPH. The results of this study indicate that in older subjects with PPH, there is a significant change in: (1) double-support time variability following glucose ingestion; and (2) gait speed following water ingestion.

Regarding SBP, as expected on the glucose only day (“G”), there was a significant decline in SBP among subjects with PPH. However, there was no significant change in SBP on the glucose day (“GW”), due to the effect of intermittent walking, which has been shown to attenuate the hypotensive effect of glucose in older subjects with PPH [37].

Gait involves the interplay between motor, sensory, visual, vestibular, cerebellar, cognitive, psychological and musculoskeletal systems [38,39,40]. It has been suggested that in hypotensive states, the accompanying reduction in cerebral perfusion pressure may cause gait unsteadiness, potentially by affecting the vestibular, cerebellar and musculoskeletal systems [41]. Furthermore, there is evidence that hypotension is associated with poorer cognitive function, in particular executive function [42], which involves the ability to plan, sequence tasks and make judgments [43]. This may be the possible mechanism by which PPH resulted in a change in double-support time variability. An increase in double-support time variability is associated with an increased falls risk in older people [17], particularly recurrent falls [17]. Therefore, this suggests an avenue by which PPH may contribute to an increased falls risk in the older person.

A reduction in gait speed is shown to be associated with an increased falls risk in older people [13,15,16,19]. Specifically, gait speed is an independent predictor of fall related femoral neck fractures [15]. Apart from the association with falls, a reduction in gait speed has also been associated with increased frailty in older people [44]. Keeping in mind that there are multiple systems influencing gait [41], the finding that there was a significant change in gait speed following water ingestion among subjects with PPH suggests that there are other mechanisms apart from hypotension that contribute to worsening of gait parameters, such as frailty. PPH could be considered more common among older people who are frail given that the prevalence of PPH is higher among older people in residential care [18,45] compared to older people residing at home [46,47]. Therefore, although frailty was not directly measured in this study, it may be possible that the older subjects with PPH were also more frail. Another factor that may have contributed to a change in gait speed among older subjects with PPH post water ingestion may be the possible effects of fasting. On the glucose day (“GW”) when compared with the water day (“WW”), fasting was not as prolonged as at *t* = 0 min subjects ingested a glucose drink before walking, whereas on the water day subjects consumed only water. To date, there have been no studies exploring the relationship between gait speed in the fasted, compared to a non-fasted, state particularly in older people who may be more vulnerable.

The lack of HR increase noted in this study is consistent with our previous finding [37] and those of others [47]. We have previously discussed [37] additional mechanisms at play that may be contributing to the development of PPH including abnormalities in the baroreceptor function [48], the age-related decline in b-adrenergic responsiveness to sympathetic activation [49,50] and concomitant usage of HR lowering medication such as beta blockers and calcium channel blockers [49,50].

While the strength of this study is that it specifically investigated the effects on gait parameters of glucose and water ingestion in older people with and without PPH, it is important to point out its limitations. Although powered to detect the primary outcome, it should be noted that the sample size was relatively small. As discussed, low-intensity intermittent walking has been suggested as a possible intervention to reduce the postprandial fall in SBP evident in people with PPH [37]. Therefore, the walking protocol may have limited the potential influence on gait parameters and future research should consider this in its design. For example, the maximum change in gait speed was noted at 60 min post drink ingestion and so it might be beneficial to undertake an investigation to study the effects of glucose ingestion on gait speed in subjects with PPH at this specific time point only. In addition, it remains unknown whether prolonged sitting has an impact on gait measurements and this will also need to be considered in future study design. As discussed, cognitive impairment and vestibular dysfunction are common in the older population and may influence gait [38,39]. Both were not measured in this study and, therefore, may have potentially affected the study outcomes. The presence of vestibular dysfunction could contribute to a decline in gait speed [51] and a decline in cognitive function is associated with an increased variability in gait parameters [52]. However, as the study recruited subjects that had previously participated in research studies or attended community events, it was anticipated that significant cognitive impairment would not be present in this study group. An assessment of muscle strength or exercise tolerance was not undertaken and may have influenced gait speed. Older people who exercise have been shown to have an increased gait speed [53,54] while reduced lower extremity strength is associated with a lower gait speed [55]. Lastly, this study highlights the challenge of undertaking gait research. In view that gait involves the coordination between multiple systems, gait assessments should include a comprehensive evaluation of factors that could influence gait. The CCI does not capture musckuloskeletal related conditions which could affect gait such as arthirits, or sensory deficits which also influence gait. Perhaps measuring frailty which is increasingly recognized as an important aspect of assessing the health status in older people [56] would have been more appropriate. The most robust gait parameter associated with frailty is a decline in gait speed [57,58,59] while an increase in stride length variability [57] has also been demonstrated to be associated with frailty.

## 5. Conclusions

This study establishes for the first time that in older people with PPH, glucose ingestion results in a change in double-support time variability, a parameter known to be associated with increased falls risk in older people. The effect of PPH on other gait parameters may have been mitigated due to the effect of intermittent walking which is known to diminish the postprandial hypotensive response.

## Figures and Tables

**Table 1:** Absolute value of declined SBP, and maximum decline and time of maximum decline in SBP following oral glucose (50 g).

PPH					No PPH				
Subject	Baseline SBP (mmHg)	Absolute Value of Declined SBP (mmHg)	Max Decline in SBP (mmHg)	Time of Max Decline in SBP (min)	Subject	Baseline SBP (mmHg)	Absolute Value of Declined SBP (mmHg)	Max Decline in SBP (mmHg)	Time of Max Decline in SBP (min)
1	145	120	25	105	1	127	113	14	60
2	169	141	28	30	2	140	129	11	36
3	145	102	43	105	3	119	107	12	90
4	136	113	23	105	4	126	123	3	18
5	153	133	20	18	5	130	119	11	42
6	134	107	27	24	6	125	113	12	18
7	166	143	23	75	7	150	134	16	36
8	158	138	20	105	8	132	118	14	60
9	144	114	30	18	9	124	113	11	30
10	173	126	47	60	10	151	135	16	18
11	145	120	25	120	11	154	151	3	18
12	132	109	23	105	12	157	143	14	54
13	133	113	20	42	13	103	95	8	30
14	136	116	20	18					
15	167	134	33	48					
16	154	134	20	36					
Mean ± SD			26.69 ± 8.43	54.0 ± 36.0				11.0 ± 3.0	42.0 ± 10.0

Abbreviations: SBP, systolic blood pressure; PPH, postprandial hypotension; max, maximum; SD, standard deviation.

**Table 2:** Subjects’ characteristics.

Characteristic	Subjects with PPH (*n* = 16)	Subjects no PPH (*n* = 13)		*p* Value
Age, years (mean ± SD)	76.5 ± 4.1		75.8 ± 6.0	0.742
Sex (*n*/%)				0.441
female	5 (31)		6 (46)	
male	11 (69)		7 (54)	
History of falls in preceding 12 months (*n*/%)	3 (19)		0	0.099
Falls Efficacy Scale International score (mean ± SD)	16.8 ± 4.8		19.5 ± 6.3	0.230
Orthostatic hypotension (*n*/%)	4 (25)		2 (15)	0.627
Autonomic dysfunction (*n*/%)	3 (19)		0	0.099
Charlson Comorbidity Index (mean ± SD)	2.0 ± 0.9		1.2 ± 0.4	0.006
Hypertension (*n*/%)	10 (63)		3 (23)	0.034
Heart disease (*n*/%)	3 (19)		1 (8)	0.390
Diabetes mellitus (*n*/%)	1 (6)		0	0.842
Hypothyroidism (*n*/%)	2 (13)		1 (8)	0.672
Antihypertensives (*n*/%)	12 (75)		4 (31)	0.017
ACEI	2 (13)		0	
ARB	7 (44)		1 (8)	
Beta Blockers	1 (6)		1 (8)	
CCB	4 (25)		3 (23)	
Diuretics	1 (6)		1 (8)	

Abbreviations: PPH, postprandial hypotension; SD, standard deviation; ACEI, angiotensin converting enzyme inhibitor; ARB, angiotensin receptor blocker; CCB, calcium channel blocker.

**Table 3:** Baseline gait parameters.

Characteristic	Subjects with PPH (*n* = 16)	Subjects No PPH (*n* = 13)	*p* Value
Gait speed “WW” day	112.9 ± 21.9	116.8 ± 27.0	0.674
Gait speed “GW” day	113.8 ± 22.0	114.4 ± 24.0	0.945
Stride length variability “WW” day	3.5 ± 1.4	2.6 ± 1.3	0.085
Stride length variability “GW” day	3.4 ± 1.4	2.6 ± 1.6	0.163
Double-support time variability “WW” day	14.1 ± 10.9	17.9 ± 17.9	0.484
Double-support time variability “GW” day	13.9 ± 12.1	17.0 ± 24.0	0.649
Swing time variability “WW” day	9.4 ± 11.7	11.4 ± 16.1	0.702
Swing time variability “GW” day	6.0 ± 6.3	15.0 ± 21.1	0.157

Abbreviations: PPH, postprandial hypotension; SD, standard deviation; SBP, systolic blood pressure; WW, water and walk; GW, glucose and walk.

## Abbreviations

The following abbreviations are used in this manuscript:

PPH	postprandial hypotension
SBP	systolic blood pressure
HR	heart rate
G	glucose
WW	water and walk
GW	glucose and walk
AUC	area under the curve
G-TRAC	Adelaide Geriatrics Training and Research with Aged Care
CCI	Charlson Comorbidity Index
max	maximum
SD	standard deviation
ACEI	Angiotensin Converting Enzyme Inhibitor
ARB	Angiotensin Receptor Blocker
CCB	Calcium Channel Blocker.
CI	confidence interval
△	change
SLV	stride length variability
DSTV	double-support time variability
STV	swing time variability
